# Better adherence to guidelines among psychiatrists providing pharmacological therapy is associated with longer work hours in patients with schizophrenia

**DOI:** 10.1038/s41537-023-00407-3

**Published:** 2023-11-07

**Authors:** Satsuki Ito, Kazutaka Ohi, Yuka Yasuda, Michiko Fujimoto, Hidenaga Yamamori, Junya Matsumoto, Kentaro Fukumoto, Fumitoshi Kodaka, Naomi Hasegawa, Keiichiro Ishimaru, Kenichiro Miura, Norio Yasui-Furukori, Ryota Hashimoto

**Affiliations:** 1 Department of Developmental and Clinical Psychology, The Division of Human Developmental Sciences, Graduate School of Humanity and Sciences, Ochanomizu University, Tokyo, Japan; 2 Department of Pathology of Mental Diseases, National Institute of Mental Health, National Center of Neurology and Psychiatry, Tokyo, Japan; 3 Department of Psychiatry, Gifu University Graduate School of Medicine, Gifu, Japan; 4 Life Grow Brilliant Mental Clinic, Medical Corporation Foster, Osaka, Japan; 5 Department of Psychiatry, Osaka University, Graduate School of Medicine, Osaka, Japan; 6 Japan Community Health Care Organization Osaka Hospital, Osaka, Japan; 7 Department of Neuropsychiatry, School of Medicine, Iwate Medical University, Iwate, Japan; 8 Department of Psychiatry, The Jikei University School of Medicine, Tokyo, Japan; 9 Faculty of Core Research, Ochanomizu University, Tokyo, Japan; 10 Department of Psychiatry, Dokkyo Medical University School of Medicine, Mibu, Japan

**Keywords:** Schizophrenia, Pharmacology

## Abstract

Schizophrenia is a psychiatric disorder that is associated with various social dysfunctions, including shorter work hours. To measure the degree to which psychiatrists adhere to guidelines for pharmacological therapy of schizophrenia, we recently developed the individual fitness score (IFS) for adherence among psychiatrists in each patient. However, it remains unclear whether better adherence among psychiatrists is associated with higher patients’ social functional outcomes, such as work hours. In this study, we examined the relationship between adherence to guidelines among psychiatrists and work hours in patients with schizophrenia. To evaluate the association between adherence to guidelines for pharmacological therapy among psychiatrists for treating schizophrenia and work hours, we used the IFS and social activity assessment, respectively, in 286 patients with schizophrenia. The correlation between IFS values and work hours was investigated in the patients. The adherence among psychiatrists to guidelines was significantly and positively correlated with work hours in patients with schizophrenia (*rho* = 0.18, *p* = 2.15 × 10^−^^3^). When we divided the patients into treatment-resistant schizophrenia (TRS) and nontreatment-resistant schizophrenia (non-TRS) groups, most patients with TRS (*n* = 40) had shorter work hours (0–15 h/week). Even after excluding patients with TRS, the positive correlation between adherence to guidelines among psychiatrists and work hours in patients with non-TRS (*n* = 246) was still significant (*rho* = 0.19, *p* = 3.32 × 10^−^^3^). We found that work hours were longer in patients who received the guideline-recommended pharmacotherapy. Our findings suggest that widespread education and training for psychiatrists may be necessary to improve functional outcomes in patients with schizophrenia.

## Introduction

Patients with schizophrenia often experience various social dysfunctions, which can strongly impact their ability to engage in social interactions and maintain relationships^1,2^. Several social dysfunctions have been observed in individuals with schizophrenia^2–4^, e.g., social withdrawal, which can be a result of various factors, including positive symptoms, negative symptoms or anxiety related to social interactions; impaired social cognition, including difficulties in recognizing facial expressions, understanding social cues or accurately perceiving others’ intentions; social anxiety, which is characterized by intense fear or discomfort in social situations; and social skills deficits, including difficulty initiating conversations, maintaining eye contact, interpreting social cues or responding appropriately in social situations. These impairments can lead to misinterpretations of social situations and difficulties in forming appropriate social relationships. The severity of social dysfunctions can vary among patients with schizophrenia. Effective treatment approaches, including pharmacological therapy, can help patients with schizophrenia manage and improve their social functioning^5,6^.

### Pharmacological therapy

Antipsychotic medications are the core of pharmacological therapy for schizophrenia^7,8^. Antipsychotic medications effectively improve mainly positive symptoms in patients with schizophrenia, and are also effective, but not as strong, in treating negative symptoms, cognitive impairment, and social dysfunction. All psychotropic medications have a risk of side effects, and it would be happened even under antipsychotic monotherapy. In addition to antipsychotic medications, other medications, such as anticholinergics, benzodiazepines, antidepressants, and mood stabilizers, may also be used as adjunctive pharmacological therapy in the treatment of specific symptoms in patients with schizophrenia, such as extrapyramidal symptoms, insomnia, and aggression; however, these adjunctive medications have further multiple potential side effects, such as constipation, sedation, cognitive impairments, medication dependence, and withdrawal symptoms^9^. The more drugs used, the higher the risk of side effects. In clinical practice, polypharmacy – i.e., the use of multiple medications concurrently—is common and a complex issue in the treatment of schizophrenia^10,11^.

### Guidelines for pharmacological therapy of Schizophrenia

Polypharmacy in schizophrenia is often based on clinical experience rather than evidence-based practice. Several national guidelines for the pharmacological treatment of schizophrenia, including the American Psychiatric Association’s Practice and the National Institute for Health and Care Excellence guidelines, have recommended that psychiatrists carefully evaluate the risks and benefits of polypharmacy vs. monotherapy as the first-line treatment options whenever possible, emphasize monotherapy and emphasize the importance of minimizing the use of multiple other medications due to the potential risks related to polypharmacy^12,13^. Similarly, the Japanese Society of Neuropsychopharmacology has published the Guidelines for Pharmacological Therapy of Schizophrenia, which also recommend that psychiatrists perform antipsychotic monotherapy without polypharmacy such as anticholinergics and benzodiazepines^14^.

### Gaps with guideline-recommended treatments and overcoming them

These clinical guidelines have been developed to standardize and improve the quality of medical care in clinical practice. These guidelines assist in making treatment strategy decisions among patients and health care professionals in clinical practice. However, the extent to which the guidelines are followed in clinical practice needs to be clarified. Studies on the gap between guidelines and clinical practice and efforts toward guideline adherence among psychiatrists are underway worldwide^15–18^. In Japan, the Effectiveness of GUIdeline for Dissemination and Education in psychiatric treatment (EGUIDE) project was launched to work toward guideline adherence among psychiatrists^19–24^. To measure the gap between guideline recommendations and actual clinical practice, we recently developed an “individual fitness score (IFS)” for each patient to evaluate whether the prescription provided by each psychiatrist adheres to the “Guideline for Pharmacological Therapy of Schizophrenia” (see Table 1 in the paper by Inada et al. ^25^). The relationships between adherence among psychiatrists to guidelines and patients’ outcomes have been reported in that patients receiving the recommended treatment have better quality and quantity of life^26^ and milder psychiatric symptoms^27^. Thus, guideline-compliant treatment would be expected to result in better functional outcomes for the patient. However, it remains unclear whether better psychiatrists’ adherence, assessed by the IFS values, is associated with higher social functional outcomes among patients, such as work hours.

### Aims

We hypothesized that longer work hours would be associated with higher IFS values, indicating better adherence to recommended pharmacological therapy for patients with schizophrenia. In this study, we examined the relationship between adherence among psychiatrists (assessed by IFS values) and social functioning (quantified by work hours) in 286 patients with schizophrenia.

## Methods

### Participants

The current study included 286 in- and outpatients with schizophrenia recruited at Osaka University Hospital. These participants fully overlapped with those in our previous studies^28–30^. Each patient with schizophrenia was diagnosed by at least two trained psychiatrists according to the criteria described in the Diagnostic and Statistical Manual of Mental Disorders, Fourth Edition (DSM-IV), based on the Structured Clinical Interview for DSM-IV (SCID). Based on the patients’ medical record information and the following criteria, we identified patients with treatment-resistant schizophrenia (TRS), whereas the other patients were treated with those with nontreatment-resistant schizophrenia (non-TRS). For patients who had been treated with clozapine or who had not been treated with clozapine but had been treated with electroconvulsive therapy (ECT), medical records were reviewed to confirm whether these patients with schizophrenia had TRS. In this study, the TRS was diagnosed according to the following definition of TRS in Japan: TRS was defined as a patient with schizophrenia who had never had a global assessment of functioning (GAF) of 41 equivalents or greater after at least four weeks of treatment with two or more appropriate doses of independent antipsychotics (chlorpromazine equivalent 600 mg/day or more, including one or more atypical antipsychotics)^31^. Therefore, to confirm whether patients had TRS, we checked (1) clozapine use or (2) ECT conducted, and if either was applicable, (3) little response (never had a global assessment of functioning: GAF of 41 equivalents or greater) when using adequate doses (chlorpromazine equivalent 600 mg/day or more) of two or more antipsychotics (including one or more atypical antipsychotics) independently for an adequate period (at least four weeks). The authors assert that all procedures contributing to this work comply with the ethical standards of the relevant national and institutional committees on human experimentation and with the Helsinki Declaration of 1975, as revised in 2008. This study was approved by the Research Ethics Committee of the National Center of Neurology and Psychiatry (Approval number: A2018-095) and Osaka University (Approval number: 706-11). Written informed consent was obtained from all participants.

### Measures

To evaluate adherence among psychiatrists to guidelines for the pharmacological therapy of schizophrenia by the Japanese Society of Neuropsychopharmacology, we calculated IFS for schizophrenia developed by Inada et al. ^25^ Because treatment recommendations in the guidelines differ whether the patient with schizophrenia has TRS or not, the formulas to calculate IFS are different for TRS and non-TRS^25^. The IFS ranges from 0 to 100 points, with 100 points indicating monotherapy with antipsychotics for non-TRS and clozapine or ECT for TRS, and points deducted for concomitant treatment with other antipsychotics or psychotropic drugs (Supplementary Fig. 1).

Work hours were assessed by psychologists and physicians using the Social Activity Assessment (SAA)^32,33^. The SAA comprises three sections: “work for pay,” “work at home,” and “student.” In each section, patients were interviewed about their working conditions over the past 12-week, and a 12-week average of hours worked per week (hr/week) was calculated. If the patient participated in more than one section, the average hours worked per week (hr/week) was added across the three sections. Even in the case of inpatients, social activities during the 12 weeks preceding the examination date were assessed.

### Statistical analysis

All statistical analyses were performed using IBM SPSS Statistics 28.0 software (IBM, Armonk, NY, USA). Since we assumed that IFS and work hours were not normally distributed using Kolmogorov‒Smirnov’s test, we performed a nonparametric test in this study. The correlation between IFS and work hours was analyzed using Spearman’s rank correlation coefficient. The significance level was set at a two-tailed *p* < 0.05.

## Results

The demographic and clinical characteristics of the patients with schizophrenia (*n* = 286) are shown in Table 1. The mean IFS ± standard deviation (SD) was 52.6 ± 39.1 (range, 0–100), and the mean work hours (hours per week) ± SD was 10.7 ± 16.3 (range, 0–82) (Table 1). The IFS has a large distribution of patients around 0 and 100 points, and the distribution of patients was clumped around 0 h of work. See scatter plots with marginal histograms in Supplementary Fig. 2.

**Table 1:** Demographic and clinical characteristics of the patients with schizophrenia (*n* = 286).

	*Mean*	*SD*	Median [Range]
Age (years)	36.0	13.2	34.0 [14–74]
Sex (male/female)	144/142		
TRS/non-TRS	40/246		
Inpatient/Outpatient	59/227		
Education (years)	13.5	2.5	13.0 [8–21]
Age at onset (years)	23.8	10.7	21.0 [6–68]
Duration of illness (years)	12.2	9.9	9.0 [0–43]
CPZeq (mg/day)	600.0	605.9	400.0 [0–3725]
Typical CPZeq (mg/day)	53.4	197.8	0 [0–1625]
Atypical CPZeq (mg/day)	546.6	550.2	400.0 [0–3725]
PANSS total	91.6	22.1	92.0 [38–175]
PANSS positive symptoms	21.0	6.0	21.0 [8–44]
PANSS negative symptoms	22.6	5.9	23.0 [9–45]
PANSS general psychopathology	48.0	11.4	48.0 [19–92]
Individual fitness score	52.6	39.0	60.0 [0–100]
Work hours (hour per week)	10.7	16.3	1.0 [0–82]

Means and standard deviations (SDs) of patients with schizophrenia are presented except for sex and TRS/non-TRS diagnosis.

*TRS* treatment-resistant schizophrenia, *CPZeq* chlorpromazine equivalent, *PANSS* positive and negative syndrome scale.

**Fig. 1:**
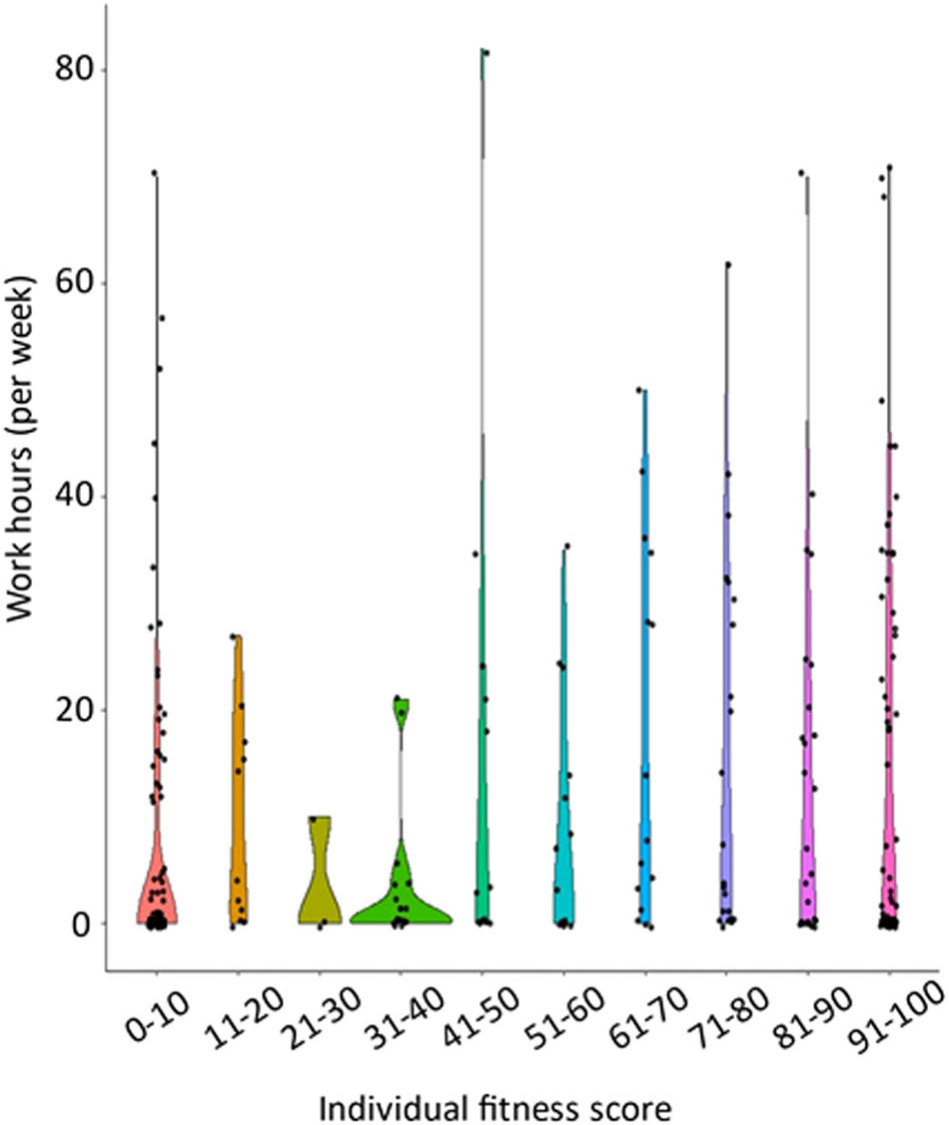
Correlation between adherence to guidelines for pharmacological therapy among psychiatrists assessed by the individual fitness score (IFS) and work hours in patients with schizophrenia. Each dot indicates the distribution of each patient with schizophrenia. Violin plots illustrate IFS in 10-point increments. A higher IFS indicates better adherence to guidelines for pharmacological therapy among psychiatrists. Work hours (per week) are calculated as a preceding 12-week average of total hours per week in patients with schizophrenia.

**Fig. 2:**
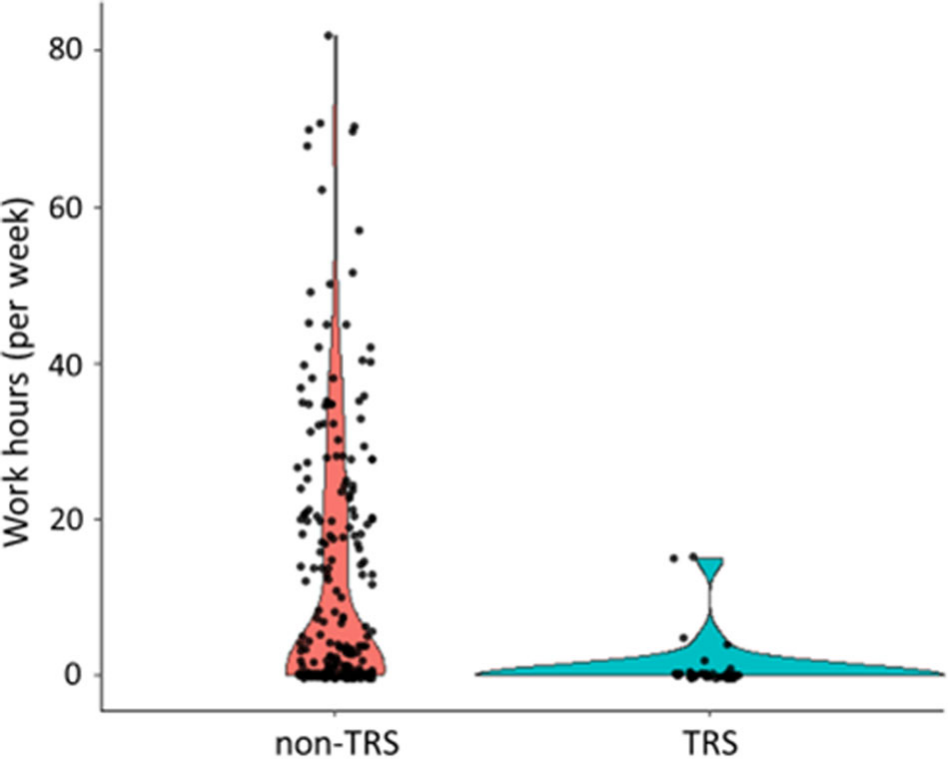
Work hours stratified by groups in treatment-resistant schizophrenia (TRS) and nontreatment-resistant schizophrenia (non-TRS). Violin plots comparing work hours for the TRS and non-TRS groups. Each dot indicates the distribution of each patient.

The adherence to guidelines for pharmacological therapy among psychiatrists of schizophrenia assessed by the IFS was significantly and positively correlated with work hours (*rho* = 0.18, *p* = 2.15 × 10^ −3^). Better adherence among psychiatrists was positively correlated with longer work hours in patients with schizophrenia (Fig. 1). Of the 286 patients with schizophrenia, there were 40 patients with TRS and 246 patients without TRS. The patients with non-TRS showed a wide range of work hours (12.2 ± 17.0, range 0–82), while most patients with TRS (34/40, 85.0%) did not work at all (all TRS patients, 1.1 ± 3.4, range 0–15) (Fig. 2). Although the number of patients with TRS in our study was limited (40/286, 14.0%), the relationship between adherence among psychiatrists and work hours might have been affected by the lack of work in patients with TRS. Thus, we investigated the relationship between adherence among psychiatrists and work hours only in patients with non-TRS (Supplementary Fig. 3). Even after restricting to patients with non-TRS, the positive correlation between adherence among psychiatrists and work hours was still significant (*rho* = 0.19, *p* = 3.32 × 10^−^^3^).

## Discussion

This study is the first to report a correlation between guideline-based drug treatment assessed by IFS values and work hours in patients with schizophrenia. We found that work hours were longer for patients receiving guideline-recommended drug treatment. As expected, patients with TRS had shorter work hours than those without TRS, and most patients with TRS did not work at all. Because we could not rule out the possibility that the correlation between the IFS values and work hours had been affected by the inclusion of the patients with TRS, we further confirmed the relationship by restricting it to patients with non-TRS. We found that the positive correlation between work hours and IFS values remained significant even after excluding patients with TRS. These results suggest that activities to promote adherence to guidelines for pharmacological therapy among psychiatrists, such as the EGUIDE project, may improve functional outcomes in patients with schizophrenia.

Patients with schizophrenia often experience difficulties in obtaining and maintaining employment, resulting in shorter hours of paid work and housework than among healthy individuals^34,35^. This can lead to significant economic burdens for both the patient and their family, as unemployment and loss of income are common consequences of the illness^36–38^. Several factors, including cognitive function, psychiatric symptoms, and social functioning, contribute to employment in patients with schizophrenia^29,39,40^. It is important to recognize that employment plays a crucial role in the lives of patients with schizophrenia. It not only provides financial stability but also has positive effects on self-esteem, reduces stigma^41^ and improves overall quality of life^28,42,43^. With appropriate support and interventions, such as vocational rehabilitation programs and job coaching, patients with schizophrenia can overcome the barriers they face and find meaningful employment. In this study, we provided the possibility that the work hours in patients with schizophrenia may be increased by these support and interventions as well as by promoting activities for increasing adherence to guidelines for pharmacological therapy among psychiatrists.

In this study, because most patients with TRS (34/40, 85.0%) did not work at all, i.e., 0 (hours per week), we investigated the relationship between work hours and adherence to guidelines for pharmacological therapy among psychiatrists for patients with non-TRS. The results indicated that better adherence among psychiatrists was positively correlated with longer work hours in patients with non-TRS. In contrast, IFS values to measure adherence among psychiatrists are calculated by separate formulas for TRS and non-TRS. Thus, it might be better to assess the relationship using a linear regression analysis with the diagnosis of TRS or non-TRS as a covariate in all patients with schizophrenia. In the analysis, we further found that the relationship between longer work hours and higher IFS values was still significant after including the TRS diagnosis as a covariate in all patients with schizophrenia (*beta* = 0.16, *p* = 4.74 × 10^−3^). Thus, these findings suggest that the relationship between work hours and adherence among psychiatrists is not restricted to patients with non-TRS but should be observed in all patients with schizophrenia.

While there are multiple quality indicators (QIs), such as the number of medications, dose, and content, to be evaluated for pharmacological therapy recommended for patients with schizophrenia, the IFS provides a comprehensive evaluation of the pharmacological therapy in each patient, which can be expressed as a single index ranging from 0 to 100 points^44^. Several reports have examined the relationship between each QI incorporated in the IFS and social functioning in patients with schizophrenia^45–47^. There have been reports that switching from polypharmacy to monotherapy improved patients’ daily living and work skills^45^ and that quality of life was higher in patients receiving typical antipsychotics than in those receiving atypical antipsychotics^46^. Clozapine treatment has also been reported to improve employment outcomes in patients with TRS^47^. In addition to these findings, our results suggest a relationship between guideline-recommended treatments and social functioning in patients with schizophrenia. To improve patients’ functional outcomes, we expect that not each QI but a comprehensive indicator including multiple QIs, such as the IFS, will be utilized more in future studies.

The relationship between guideline adherence among psychiatrists and patient work hours in patients with non-TRS might have been influenced by confounding and clinical factors, such as age, sex, years of education, age at onset, duration of illness, and symptom severity. Therefore, we explored the influences of these confounding and clinical factors on the relationship using regression analyses with each factor as a covariate. The relationship between psychiatrists’ adherence and patient work hours remained significant even after correcting for these factors (*p* < 0.05), except for negative symptoms (beta = 0.054, *p* = 0.064). Since previous studies have reported that working hours and psychiatrists’ adherence to guidelines are associated with negative symptoms, respectively^28,44^, our findings might have been affected by negative symptom severity.

There are some limitations to interpreting our findings. We obtained the prescription details at the assessment of work hours, and calculated the IFS based on the prescription at the point. Thus, this study did not capture how long patients have been taking the same medication, or histories of the prescriptions and treatment response. We did not have information about social background. The patient’s social background might affect our findings. Furthermore, our study design was cross-sectional. Therefore, we could not assess a true causal relationship between guideline adherence among psychiatrists and patient work hours at the study design. To conclude the causal relationship, further longitudinal study in a controlled social background situation is required. To increase working hours, pharmacological therapy as well as psychosocial interventions or family support are important factors^1,7,48^. However, we did not have information about psychosocial intervention or family support in our patients. Future studies should consider these effects on the relationship between guideline adherence among psychiatrists and working hours. Work hours have been reported to be affected by cognitive dysfunction^29^. Cognitive dysfunction, as well as psychiatric symptoms, may mediate the relationship between high IFS and work hours. The SAA assesses three domains: Work for Pay, Work at Home, and Student sections^32^. This study used the sum of these subitems to measure work hours. As the required skills differ among the three sections, it is possible to examine the relationship with adherence among psychiatrists by further subdividing work hours. Several studies have reported that antipsychotic polypharmacy with clozapine and other antipsychotics is superior to monotherapy of clozapine^49,50^. However, the current Japanese clozapine prescribing system does not permit combination therapy with clozapine and other antipsychotics. Therefore, polypharmacy with clozapine and antipsychotics is not recommended in the Japanese guidelines at this time. If the Japanese prescribing system and guidelines are updated in the future, we will need to update the IFS calculation method. In this study, according to the Japanese criteria for TRS, we defined the TRS. However, the use of internationally recognized criteria^51^ might be more appropriate.

In conclusion, we found that better psychiatrists’ comprehensive adherence to guidelines for pharmacological therapy for schizophrenia was correlated with longer work hours in patients with schizophrenia. To improve functional outcomes in patients with schizophrenia, further widespread education and training for the guidelines for psychiatrists, such as the EGUIDE project, would be required to increase psychiatrists’ comprehensive adherence to the guidelines.

### Supplementary information

Supplementary Information

## Data Availability

Study data will not be shared with the public because participants have not consented to public access. However, data supporting the results of this study will be provided upon reasonable request to the Corresponding Author (K.O., ohi.kazutaka.h8@f.gifu-u.ac.jp).
