# Supplementary material for: Better adherence to guidelines among psychiatrists providing pharmacological therapy is associated with longer work hours in patients with schizophrenia
Source: Schizophrenia (Heidelb). 2023 Nov 7;9(1):78. doi: 10.1038/s41537-023-00407-3 (PMC10630392; doi:10.1038/s41537-023-00407-3)

**Supplementary Figure 1.** Flowchart of the procedure for calculating the individual fitness score (IFS). The IFS is calculated by subtracting the points corresponding to each item from the 100-point scale. A score of 0 is considered zero if the item is below 0. If there is no corresponding arrow, the point reduction stops and proceeds to the item below the plus sign at the bottom. For example, if monotherapy is "yes," the SGA is applicable, and "less than maximum dose" is also "yes," there is no "yes" arrow on the flow chart, and the point reduction stops there. Then, proceed to the cell for point reduction for concomitant use other than antipsychotics. Abbreviations: CPZ, chlorpromazine: ECT, electroconvulsive therapy: FGA, first-generation antipsychotic: non-TRS, nontreatment-resistant schizophrenia: SGA, second-generation antipsychotic: TRS, treatment-resistant schizophrenia.

**
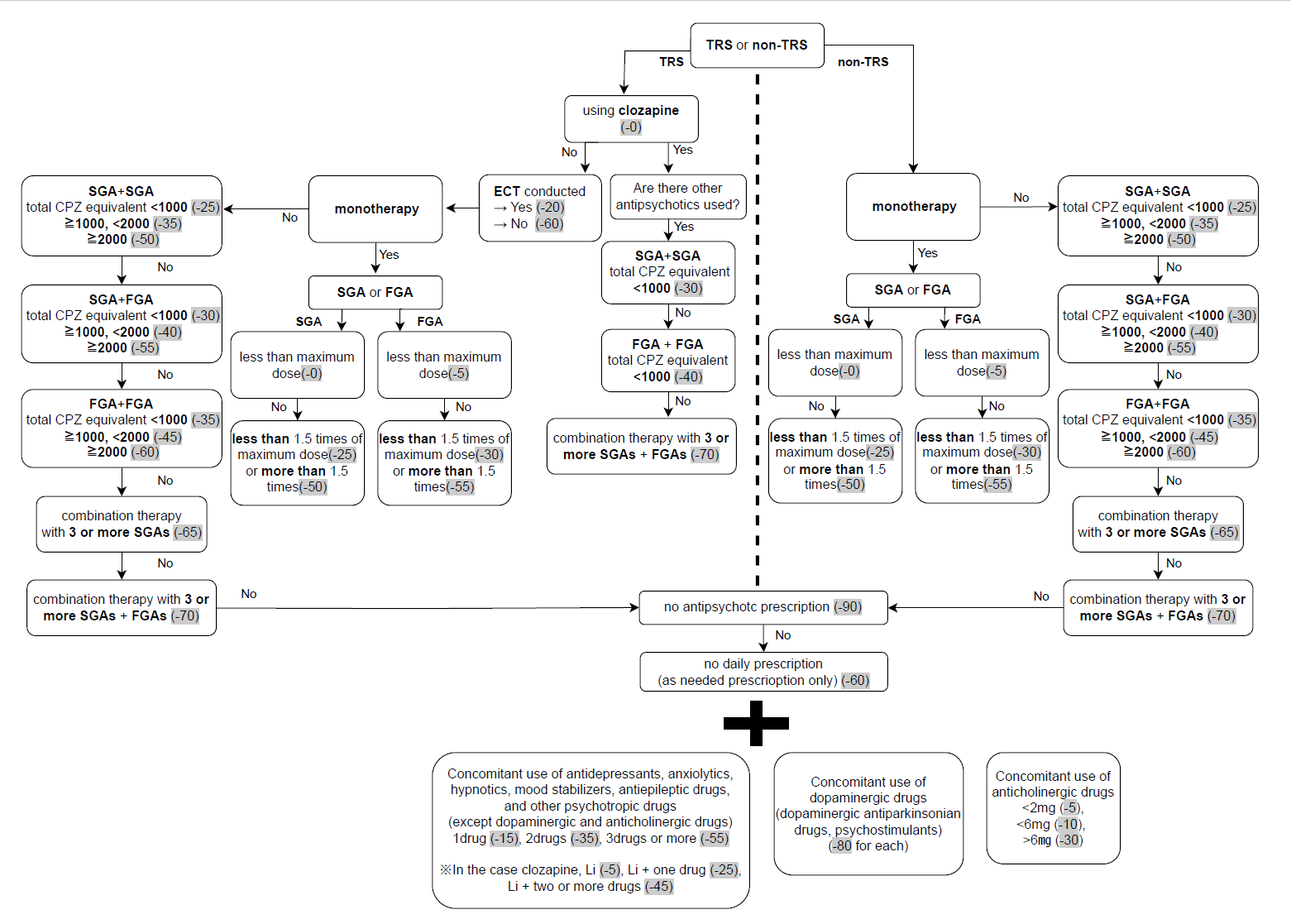
**

**Supplementary Figure 2.** Scatter plots showing the relationship between IFS and working hours and marginal histograms showing their distribution.

**
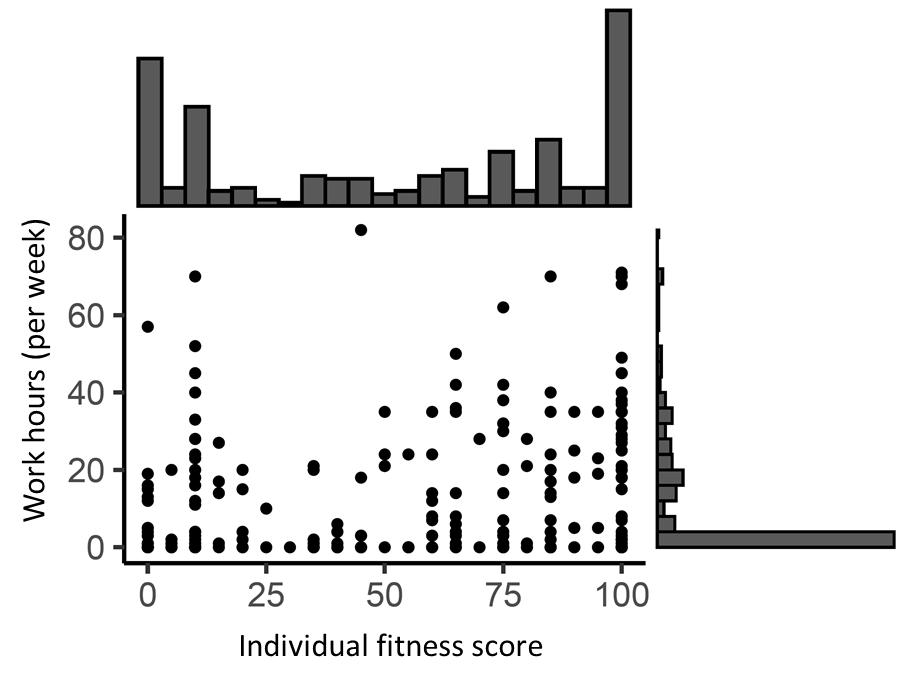
**

**Supplementary Figure 3.** Correlation between adherence to guidelines for pharmacological therapy among psychiatrists assessed by IFS and work hours in patients with non-treatment-resistant schizophrenia (non-TRS). Each dot indicates the distribution of each patient with non-TRS. Violin plots illustrate IFS in 10-point increments.


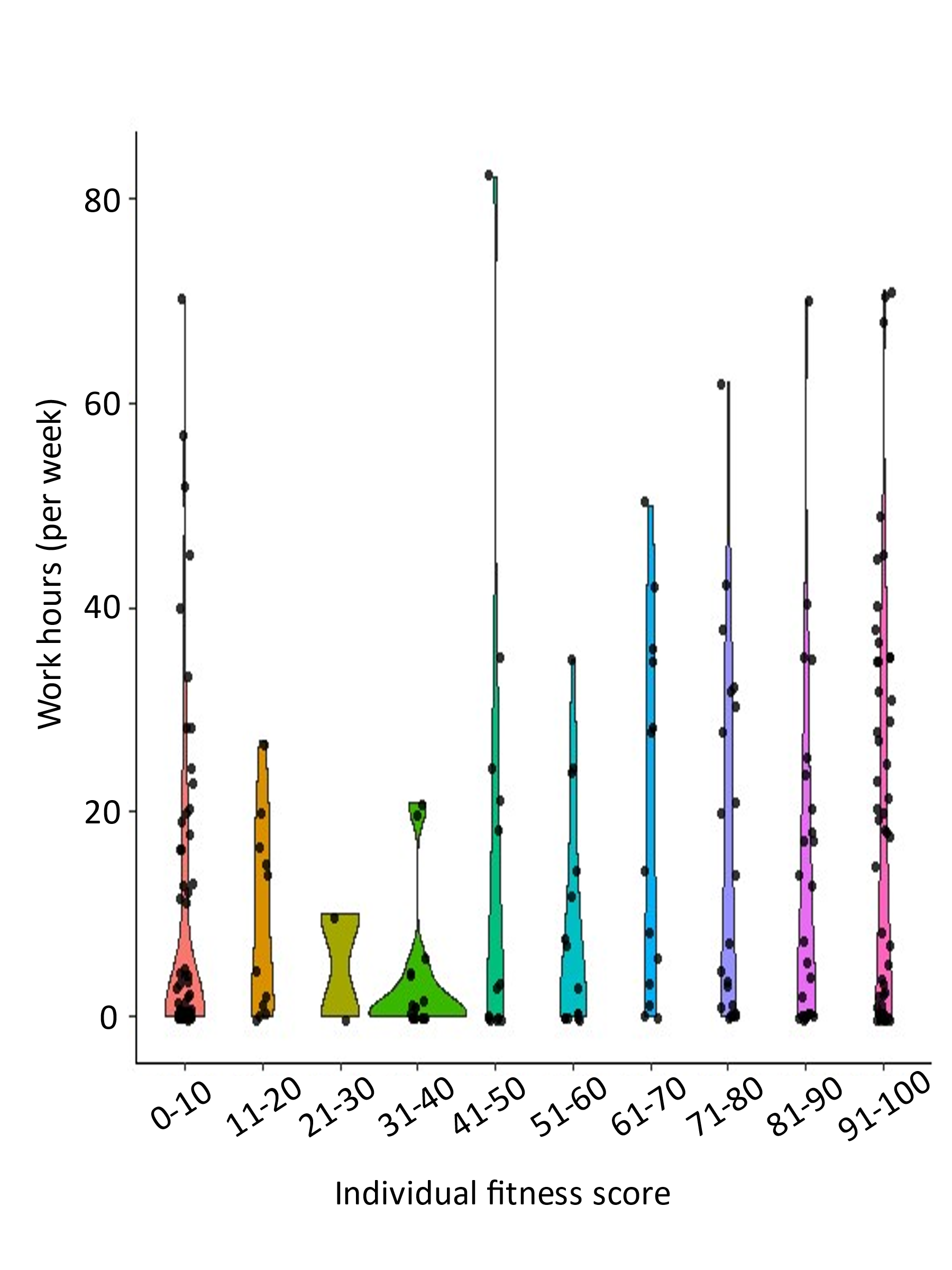

Supplement: Supplementary file 1 — Supplementary Information [file 41537_2023_407_MOESM1_ESM.docx]
